# *Colibacter massiliensis* gen. nov. sp. nov., a novel Gram-stain-positive anaerobic diplococcal bacterium, isolated from the human left colon

**DOI:** 10.1038/s41598-019-53791-1

**Published:** 2019-11-20

**Authors:** Hussein Anani, Rita Abou Abdallah, May Khoder, Anthony Fontanini, Morgane Mailhe, Davide Ricaboni, Didier Raoult, Pierre-Edouard Fournier

**Affiliations:** 1 Aix Marseille Univ, Institut de Recherche pour le Développement (IRD), Service de Santé des Armées, AP-HM, UMR Vecteurs Infections Tropicales et Méditeranéennes (VITROME), Marseille, France; 2 Institut Hospitalo-Universitaire Méditerranée-Infection, Marseille, France; 3 Aix-Marseille Université, Institut de Recherche pour le Développement (IRD), UMR Microbes Evolution Phylogeny and Infections (MEPHI), Marseille, France; 4 Special Infectious Agents Unit, King Fahd Medical Research Center, King Abdulaziz University, Jeddah, Saudi Arabia

**Keywords:** Bacterial genomics, Clinical microbiology

## Abstract

The gut microbiota is considered to play a key role in human health. As a consequence, deciphering its microbial diversity is mandatory. A polyphasic taxonogenomic strategy based on the combination of phenotypic and genomic analyses was used to characterize a new bacterium, strain Marseille-P2911. This strain was isolated from a left colon sample of a 60-year old man who underwent a colonoscopy for an etiological investigation of iron-deficiency anemia in Marseille, France. On the basis of 16S rRNA sequence comparison, the closest phylogenetic neighbor was *Anaeroglobus geminatus* (94.59% 16S rRNA gene sequence similarity) within the family Veillonellaceae. Cells were anaerobic, Gram-stain-positive, non-spore-forming, catalase/oxidase negative cocci grouped in pairs. The bacterium was able to grow at 37 °C after 2 days of incubation. Strain Marseille-P2911 exhibited a genome size of 1,715,864-bp with a 50.2% G + C content, and digital DNA-DNA hybridization (dDDH) and OrthoANI values with *A. geminatus* of only 19.1 ± 4.5% and 74.42%, respectively. The latter value being lower than the threshold for genus delineation (80.5%), we propose the creation of the new genus *Colibacter* gen. nov., with strain Marseille-P2911^T^ (=DSM 103304 = CSUR P2911) being the type strain of the new species *Colibacter massiliensis* gen. nov., sp. nov.

## Introduction

With 10^11^ to 10^12^ cells per gram^1^, the commensal microbiota that resides in the colon has become a focus of interest that is attracting the attention of scientists. Although this complex flora plays a role in homeostasis, it has been demonstrated to participate in triggering intestinal and extra-intestinal diseases in sensitive people^2^. The microbial diversity of the colon microbiota may result from a co-evolution with its host^3^ but may be affected by environmental conditions^4^. In order to identify all bacteria (including uncultured and fastidious) present in the colon, we use the culturomics strategy based on diversified culture conditions (temperature, media, and atmosphere), and rapid bacterial identification using matrix-assisted desorption ionization-time of flight mass spectrometry (MALDI-TOF MS)^5–7^. For putative new taxa, we use the taxonogenomic method that combines phenotypic characteristics and whole genome sequencing analysis to describe new bacterial species^8–10^. In 2016, we isolated the new bacterial strain Marseille-P2911 (=CSUR P2911 = DSM 103304), from a left colon sample of a 60-year-old patient who underwent a colonoscopy for the etiological investigation of iron-deficiency anemia in Marseille, France^11^. This bacterium was identified by matrix-assisted desorption ionization–time of flight mass spectrometry (MALDI-TOF MS) using a Microflex spectrometer^12^ (Bruker Daltonics, Bremen, Germany). The strain was predicted to be affiliated with members of the family of Veillonellaceae but distinct form species with a validly published name. In the present study, we aimed at comparing strain Marseille-P2911 to its closely related phylogenetic neighbors, and at proposing the creation of the new genus *Colibacter massiliensis* gen. nov., sp. nov.

## Results

### Strain identification and classification

**Figure 1:**
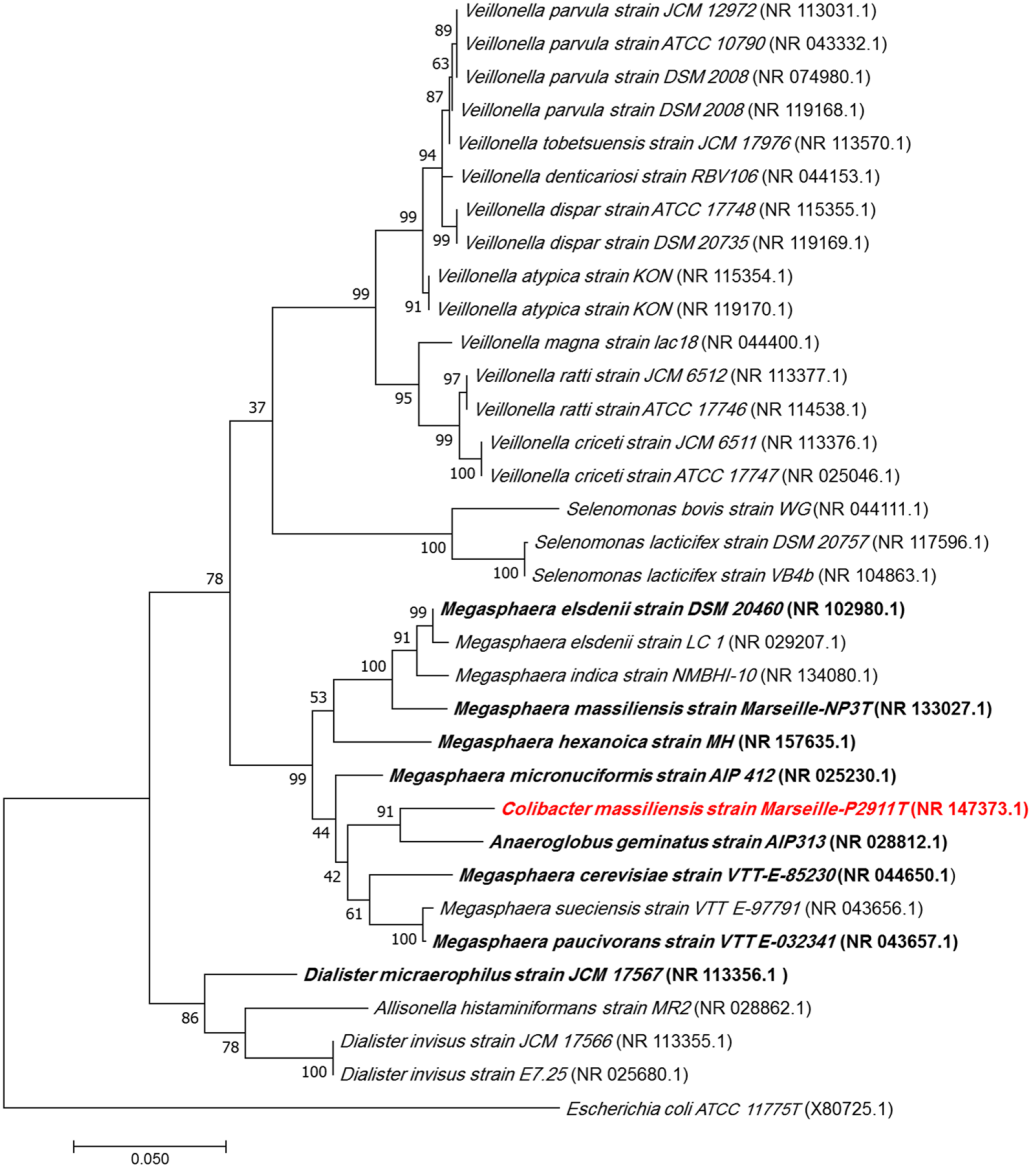
Phylogenetic tree highlighting the position of *Colibacter massiliensis* (in red) relative to other closely related bacterial taxa. Strains in bold are the eight closely related bacterial type strains with available genomes. The sequence of *Escherichia coli* ATCC 11775 served as outgroup. Genbank accession numbers for each 16s rRNA gene sequences are presented in parentheses. Sequences were here aligned by Muscle v3.8.31 with default parameters and finally 1247 nucleotides were considered to infer the phylogenetic relationship, using the Maximum Likelihood method with 1000 bootstrap replicates, within MEGA 7 software version 7.0.

Strain Marseille-P2911 was isolated from the left colon liquid sample of a 60-year-old man who underwent a colonoscopy for the etiological investigation of an iron-deficiency anemia. The patient provided signed informed consent, and the study was approved by the ethics committee of the Institut Fédératif de Recherche IFR48 under number 2016-010. Strain Marseille-P2911 could not be identified by our systematic MALDI-TOF MS screening as the score was lower than 1.8, suggesting that the corresponding species was not in the database (Fig. S1). Moreover, strain Marseille-P2911 exhibited a 94.59% 16S rRNA sequence similarity with *Anaeroglobus geminatus* strain AIP 313.00 (GenBank accession no. AF338413), the phylogenetically-closest species with standing in nomenclature (Fig. 1). As this value is lower than the 95% threshold proposed by Stackebrandt and Ebers for defining a new genus, strain Marseille-P2911 was considered as a reprsentative of a putatively new genus within the family Veillonellaceae in the phylum Firmicutes.

### Phenotypic characteristics

**Figure 2:**
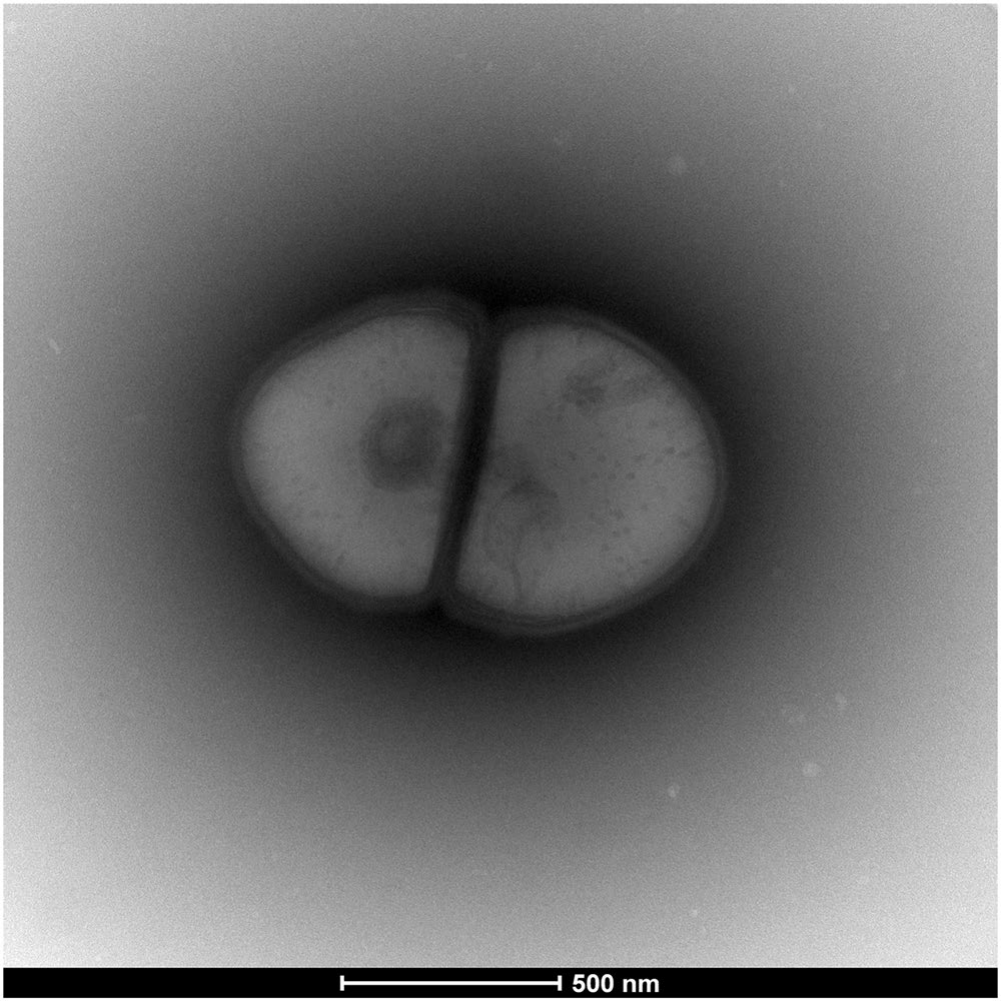
Transmission electron microscopy of *Colibacter massiliensis* using a Technai G2 electon microscope operating at 60 Kv (the scale bar represents 500 nm).

Growth was observed on 5% sheep blood-enriched Columbia agar (BioMérieux) at 37 °C after 2 days of incubation. Colonies from strain Marseille-P2911 showed neither pigmentation nor haemolysis. They were circular with a diameter of 0.1 mm, and transparent. Bacterial cells were Gram-positive, non-motile diplococci with a diameter of 0.4 to 0.6 µm, as determined by transmission electron microscopy (Fig. 2). Strain Marseille-P2911 grew only in anaerobic conditions. The sporulation test (20 minutes at 80 °C) was negative. In addition, this bacterium had neither oxidase nor catalase activities. Biochemical properties from strain Marseille-P2911, as determined by the use of API ZYM, API 20E and API 50 CH strips (bioMérieux), are detailed below in the description of *Colibacter massiliensis* gen. nov., sp. nov. paragraph.

According to the French Microbiology Society^13^, susceptibility tests showed that strain Marseille-P2911 was resistant to penicillin, piperacillin/tazobactam, ticarcillin-clavulanic acid, cefoxitin, ceftriaxone, ciprofloxacin, fosfomycin, trimethoprim-sulfamethoxazole, metronidazole, rifampin, amikacin and teicoplanin.

By comparison with closely related taxa, strain Marseille-P2911 differed in a combination of negative oxidase activity and positive sucrose/raffinose metabolism (Table 1). The most abundant fatty acids were saturated ones (83%), including dodecanoic acid that represented 25% of all fatty acids extracted from the bacterial cell wall. However, a specific 14:0 3-OH and several branched fatty acids were also described (Table S1).

**Table 1:** Compared phenotypic characteristics of studied species.

Characteristics	CM	AG	DM	MP	MMi	MC	ME	MMa	MH
Gram stain	+	−	−	−	−	−	−	−	−
Production of Catalase	−	−	−	−	−	−	−	−	−
Oxidase	−	−	−	−	−	−	−	+	−
Nitrate reductase	na	−	−	−	−	−	−	na	−
Gelatin hydrolysis	+	−	−	−	−	−	+	+	−
Utilisation of L-Arabinose	−	−	−	−	−	±	−	+	−
D-Galactose	+	+	−	−	−	−	−	+	w
D-Fructose	+	−	−	−	−	+	+	+	+
D-Mannose	+	+	−	−	−	−	−	+	±
D-Rhamnose	na	−	−	−	−	−	−	+	−
D-Mannitol	+	−	−	−	−	−	+	+	−
Salicin	+	−	−	−	−	−	−	+	−
D-Cellobiose	+	−	−	−	−	−	−	+	−
D-Maltose	+	−	−	−	−	−	+	+	−
D-Lactose	+	−	−	−	−	−	−	+	−
D-Trehalose	+	−	−	na	na	na	na	+	−
Inulin	−	+	na	−	+	−	−	na	−
Starch	−	−	−	na	na	na	na	na	−
Glycogen	−	+	na	−	+	−	−	na	−
D-Glucose	+	−	−	−	−	−	+	+	−
Raffinose	+	−	−	−	−	−	−	−	−
D-Xylose	−	−	−	−	−	−	−	+	−
Sucrose	+	−	−	na	−	na	−	−	−

Differential characteristics of *Colibacter massiliensis*^11^ *(CM)*, *Anaeroglobus geminatus*^37^ *(AG), Dialister micraerophilus*^38^ *(DM), Megasphaera paucivorans*^39^ *(MP), Megasphaera micronuciformis*^40^ *(MMi), Megasphaera cerevisiae*^41,42^ *(MC), Megasphaera elsdenii*^42,43^ *(ME), Megasphaera massiliensis*^44^ *(MMa)* and *Megasphaera hexanoica*^45^ *(MH)*. +positive reaction, −negative reaction, ±variable, w week reaction, na data not available.

### Genome sequencing information and genome properties

**Figure 3:**
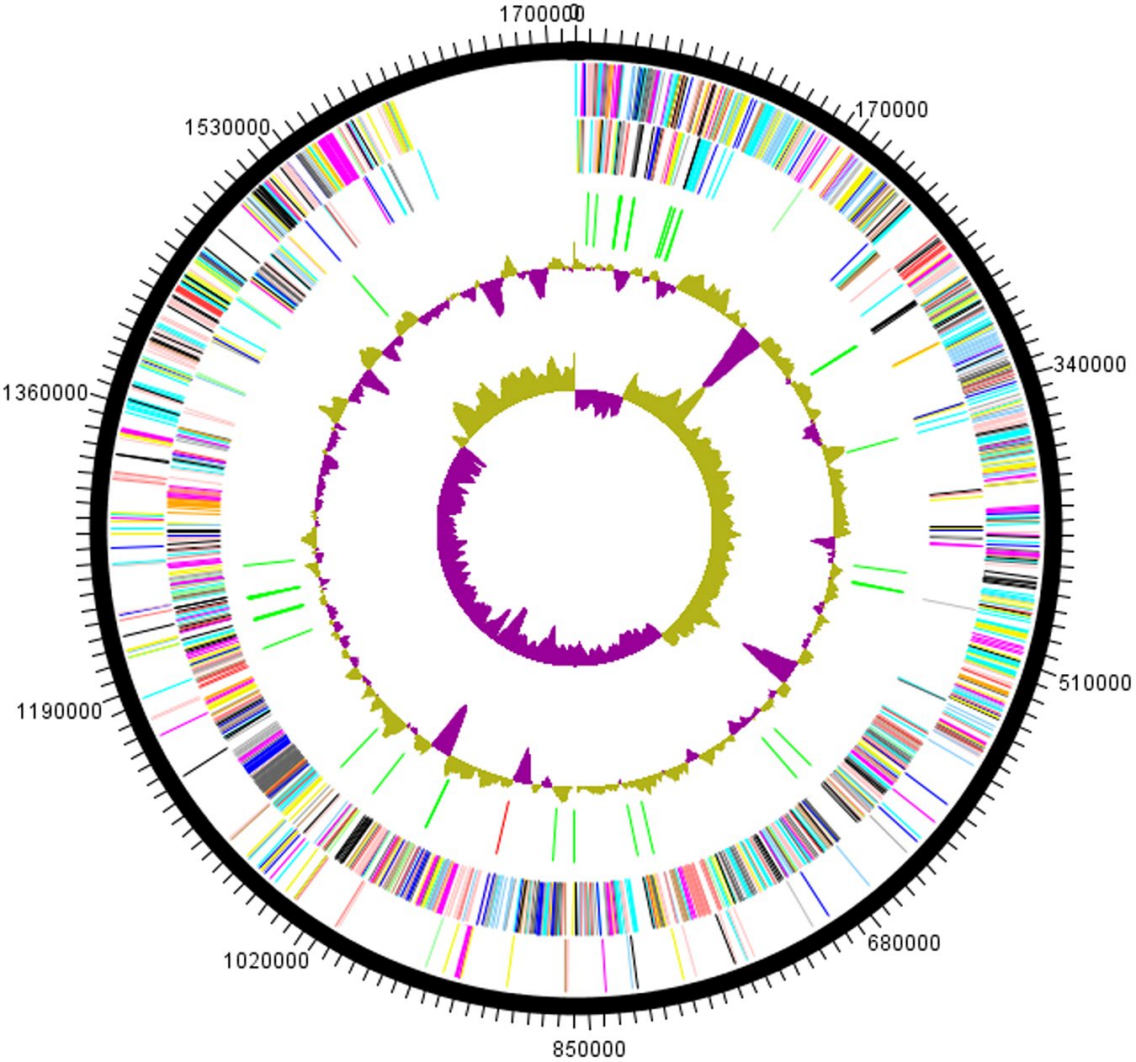
Graphical circular map of the genome. From outside to the center: genes on the forward strand colored by Clusters of Orthologous Groups of proteins (COG) categories (only genes assigned to COG). Genes on the reverse strand colored by COG categories (only genes assigned to COG). RNA genes (tRNAs green, rRNA red). G + C content and G + C skew.

**Figure 4:**
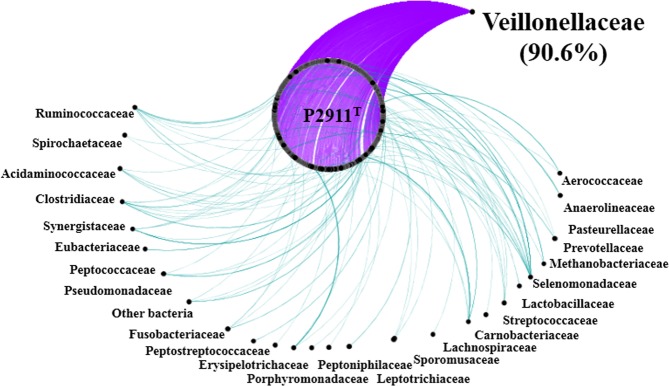
Network showing the origin of genes in *C. massiliensis* according to the bacterial families.

The genome size of strain Marseille-P2911 was 1,715,864 bp long with a 50.2% G + C content. It was assembled into 2 scaffolds. Of the 1,655 predicted genes, 1,567 were protein-coding genes and 62 were RNAs (one complete rRNA operon, three additionnal 5S rRNAs and 49 tRNA genes). A total of 1,350 genes (81.57%) were assigned a putative function (by COGs) and 305 genes (18.43%) were annotated as hypothetical proteins. The genome properties and distribution of genes into COGs functional categories are detailed in Table S2 and Fig. 3. Genes putatively gained by hypothetical lateral gene transfer (LGT) were classified according to the bacterial families of origin (Fig. 4). Although we cannot rule out the possibility that some of the transfers may be from as yet unidentified taxa, most hypothetical lateral gene transfer (LGT)-acquired genes were obtained from members of the Veillonellaceae (90.6%) and Selenomonadaceae (1.6%) families.

### Comparison with closely related bacterial strains

**Figure 5:**
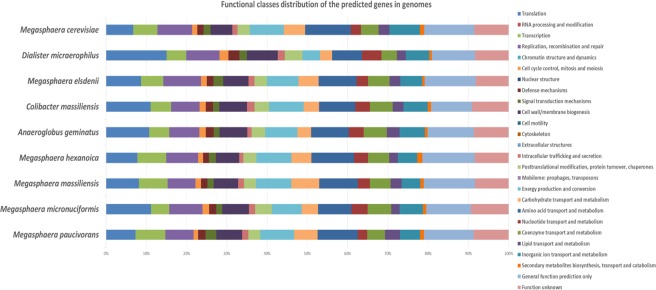
Distribution of functional classes of predicted genes according to the clusters of orthologous groups of proteins of *Colibacter massiliensis* among other bacterial taxa type strain.

**Figure 6:**
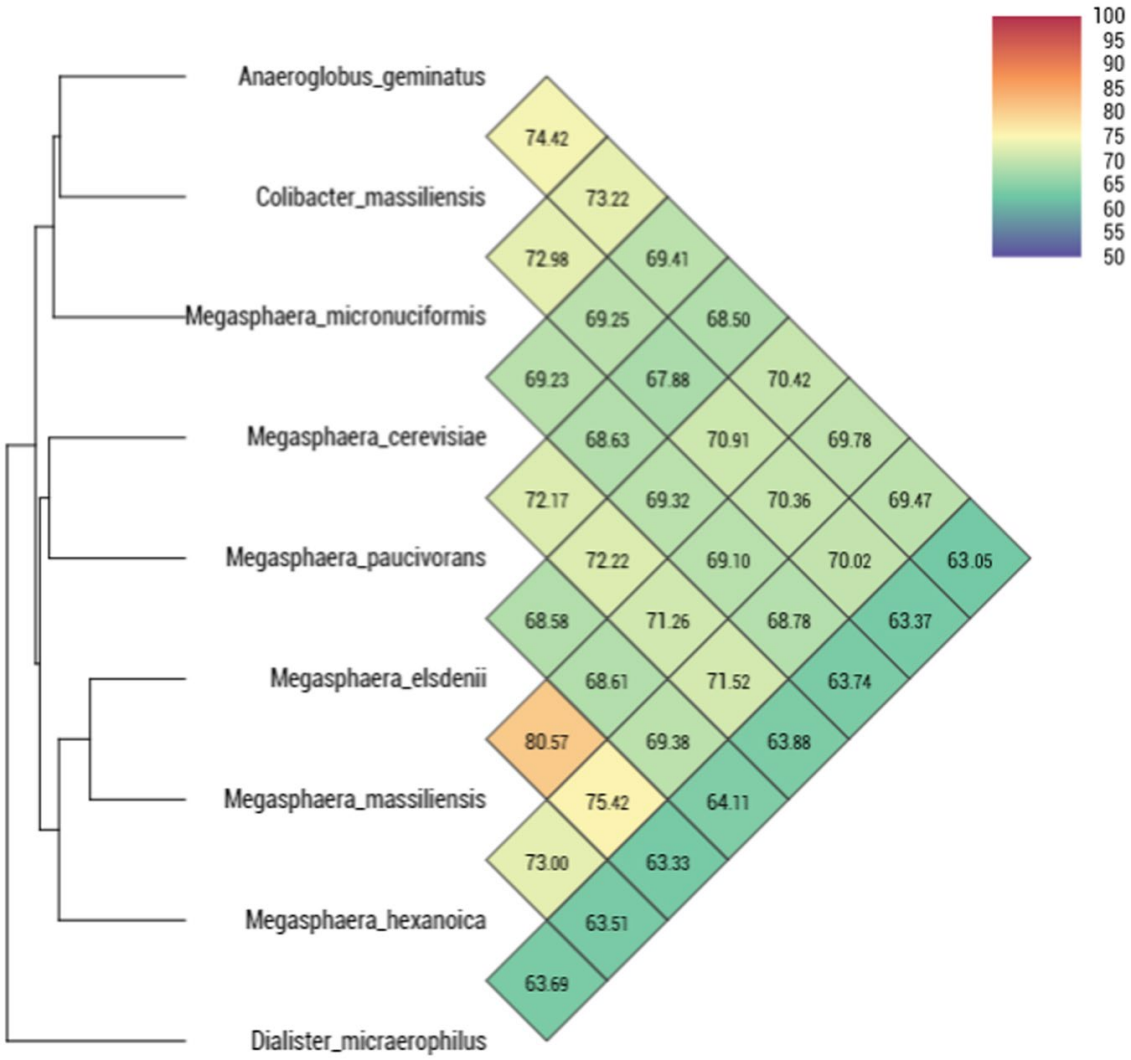
Heatmap generated with OrthoANI values calculated using the OAT software between *Colibacter massiliensis* and other closely related taxa with standing in nomenclature.

The genome of strain Marseille-P2911 was compared to the available genomes of eight closely related bacterial type strains. This comparison revealed that the genome size of our strain (1.72 Mb) was larger than that of *Dialister micraerophilus* (1.28 Mb), but smaller than those of *Anaeroglobus geminatus, Megasphaera cerevisiae, M. elsdenii, M. hexanoica, M. massiliensis, M.micronuciformis* and *M. paucivorans* (1.8, 3.24, 2.5, 2.88, 2.74, 1.77 and 2.91 Mb, respectively) (Table S3). The G + C content of strain Marseille-P2911 (50.2 mol %) was equal to that of *M. massiliensis*, but greater than those of all compared species (Table S3) except *Megasphaera elsdenii* (52.8%). The gene content of strain Marseille-P2911 (1,655) was similar to that of *D. micraerophilus* but smaller than those of other compared genomes (Table S3). The distribution of genes into COG categories was similar in all nine compared genomes (Fig. 5). Strain Marseille-P2911 shared 1169, 1161, 1129, 1109, 1083, 1081, 1071 and 666 orthologous genes with *M. micronuciformis, M. cerevisiae, M. hexanoica, M. massiliensis, A. geminatus, M. elsdenii, M. paucivorans* and *D. micraerophilus*, respectively (Table 2). Moreover, the MAGi analysis showed that AGIOS (Average Genomic Identity of Orthologous gene Sequences) values ranged from 47.76% between *M. elsdenii* and *D. micraerophilus*, to 50.93% between *M. paucivorans* and *D. micraerophilus*, among studied bacterial strains. Regarding strain Marseille-P2911, AGIOS values ranged from 48.86% with *D. micraerophilus* to 50.78% with *A. geminatus* (Table 2). Using dDDH analysis, strain Marseille-P2911 exhibited values ranging from 18.1% with *M. massiliensis* to 27.1% with *D. micraerophilus* (Table S4). These values are lower than the 70% threshold used for delineating prokaryotic species, thus confirming that this strain represents a new species. Finally, strain Marseille-P2911 exhibited average nucleotide identity (ANI) values ranging from 63.37% with *D. micraerophilus* to 74.42% with *A. geminatus*. An ANI value lower than 80.5% suggesting that two strains belong to distinct genera, we considered that strain Marseille-P2911 was representative of a new genus (Fig. 6). Consequently, based on the presented phenotypic and genomic data, we propose the creation of the new genus *Colibacter* gen. nov., with strain Marseille-P2911^T^ being the type strain of the novel species *Colibacter massiliensis* gen. nov., sp. nov.

**Table 2:** Numbers of orthologous proteins shared between genomes (upper right) and AGIOS values (lower left). The numbers of proteins per genome are indicated in bold.

	MP	MMi	MMa	MH	AG	CM	ME	DM	MC
MP	**2717**	1089	1309	1340	972	1071	1300	672	1418
MMi	50.45	**1745**	1154	1162	1090	1169	1124	665	1201
MMa	49.55	50.37	**2536**	1405	1029	1109	1441	675	1444
MH	50.26	50.18	50.6	**2785**	1019	1129	1367	692	1442
AG	49.85	50.59	50.39	50.58	**1969**	1083	1003	594	1075
CM	49.81	50.42	50.46	50.31	50.78	**1661**	1081	666	1161
ME	48.97	49.83	50.78	50.42	50.4	50.38	**2333**	683	1434
DM	50.93	50.1	48.48	49.22	49.07	48.86	47.76	**1245**	683
MC	50.61	50.47	50.08	50.3	50.41	50.08	49.79	50.18	**3086**

*Colibacter massiliensis (CM)*, *Anaeroglobus geminatus (AG), Dialister micraerophilus (DM), Megasphaera paucivorans (MP), Megasphaera micronuciformis (MMi), Megasphaera cerevisiae (MC), Megasphaera elsdenii (ME), Megasphaera massiliensis (MMa)* and *Megasphaera hexanoica (MH)*.

## Discussion

The gastrointestinal tract harbors a complex microbial microflora whose dynamic composition is important for health^14^. Here we aimed at describing a new bacterial species to enrich the knowledge on the human microbiome, using the culturomics and taxonogenomic strategies^7,10,15^.

The phylogenetic and phenotypic analysis of the new strain Marseille-P2911 revealed several distinct traits when compared to other members of the family Veillonellaceae^16^, suggesting that it could be classified in a new species of a new genus. The family Veillonellaceae is currently made of six genera of Gram-negative bacteria, including *Veillonella* (twelve species), *Megasphaera* and *Dialister* (five species each), *Allisonella*, *Anaeroglobus* and *Negativicoccus* (one species each)^17^.

As strain Marseille-P2911, many members of the family Veillonellaceae were detected in humans. For example, *Allisonella histaminiformans* was previously isolated from the vagina and *A. geminatus* was isolated from the gastrointestinal tract, stool and skin^17^. Moreover, these two species were demonstrated to be pathogenic in several diseases such as community-acquired pneumonia and advanced caries^17^.

The genomic content of strain Marseille-P2911 (dDDH, orthoANI, and AGIOS values) comforted its new species status. We observed a significant similarity to genes from the family Veillonellaceae (90.6%) (Fig. 4). A small rate of hypothetical lateral gene transfer (9.4%) from other bacterial families was observed, notably several toxin/antitoxin system-related genes putatively acquired from the families Eubacteriaceae, Lachnospiraceae, Lactobacillaceae, Selenomonadaceae and Streptococcaceae). So, we formally propose the creation of the new genus and species *Colibacter massiliensis* gen. nov., sp. nov., within the family Veillonellaceae. The type strain, Marseille-P2911^T^, was deposited in the DSMZ and CSUR collections under accession numbers DSM 103304 and CSUR P2911, respectively. The 16S rRNA and genome sequences are available in GenBank under accession numbers LT576403 and FMIY00000000, respectively. The Digital Protologue TaxoNumbers (http://imedea.uibcsic.es/dprotologue/index.php) of *C. massiliensis* gen. nov., sp. nov. is GA00103.

### Description of *Colibacter* gen. nov

*Colibacter* (Co.li.bac’ter, N.L. masc. n. *colibacter*, composed of colon, the organ from which the strain was first isolated, and bacter, a rod. *Colibacter*, a bacterium from the colon). Cells are diplococci, anaerobic, Gram-positive, nonmotile, asporogenous, catalase and oxidase negative.

### Description of *Colibacter massiliensis* gen. nov., sp. nov

*Colibacter massiliensis* (mas.si.li.en’sis; L. fem. adj. *massiliensis*, for Massilia, the Roman name of Marseille, where the type strain was first isolated).

In addition to the description features of the genus, cells have a diameter varying from 0.4 to 0.6 µm. Colonies grown on 5% sheep blood-enriched Columbia agar (bioMérieux) are circular and transparent after 2 days of incubation in anaerobic atmosphere. Growth occurs at 37 °C. Cells grow anaerobically only. Using an API ZYM strip, a positive reaction was observed for alkaline phosphatase, esterase (C4), lipase esterase (C8), leucine arylamidase, valine arylamidase, cystine arylamidase, α-chymotrypsine, phosphatase acid, naphtol-AS-BI-phosphohydrolase, ß-galactosidase, α-glucosidase, ß-glucosidase and N-acetyl-ß-glucosaminidase, but negative reactions were obtained for lipase, trypsin, α-galactosidase, ß-glucuronidase, α-mannosidase and α-fucosidase. Using an API 20E strip, a positive reaction were obtained for ß-galactosidase, arginine dihydrolase, urea and gelatin hydrolysis, glucose fermentation, mannitol, inositol, sorbitol, rhamnose, sucrose, melibiose, amygdalin and arabinose. Using an API 50 CH strip, a positive result was shown for D-ribose, D-galactose, D-glucose, D-fructose, D-mannose, D-mannitol, D-sorbitol, N-acethylglucosamine, amygdalin, arbutin, esculin iron citrate, salicin, D-celibiose, D-maltose, D-lactose, D-melibiose, D-saccharose, D-trehalose, D-melezitose, D-raffinose and D-tagatose. In contrast, negative reactions were observed for glycerol, methyl-αD-glucopyranoside, D-turanose, potassium 5-Ketogluconate, erythritol, D-arabinose, L-arabinose, D-xylose, L-xylose, D-adonitol, methyl-ßD-xylopyranoside, L-sorbose, L-rhamnose, dulcitol, inositol, methyl-αD-mannopyranoside, inulin, starch, glycogen, xylitol, gentiobiose, D-lyxose, D-fucose, L-fucose, D-arabitol, L-arabitol, potassium gluconate and potassium 2-ketogluconate. The major fatty acid found for this strain was dodecanoic acid. The genome is 1,715,864 bp long with 50.2% G + C content.

The type strain, Marseille-P2911^T^, isolated from the left colon of a patient, was deposited in the DSMZ and CSUR collections under accession numbers DSM 103304 and CSUR P2911, respectively. The 16S rRNA and genome sequences are available in GenBank under accession numbers LT576403 and FMIY00000000, respectively.

## Materials and Methods

### Strain isolation and phenotypic tests

The left colon liquid sample of a 60-year-old-man, who underwent a colonoscopy for an aetiological investigation of iron-deficiency anemia, was initially collected in La Timone Hospital in Marseille, France. From this sample, strain Marseille-P2911 was isolated after 3 days of preincubation in an anaerobic blood culture bottle (VersaTREK REDOX 2, Thermo Scientific, Villebon sur Yvette, France) supplemented with 5 mL of 0.2 µm-filtered rumen. This enriched liquid medium was then inoculated on 5% sheep blood-enriched Columbia agar (BioMérieux, Marcy l’Etoile, France) followed by an incubation at 37 °C in anaerobic atmosphere (AnaeroGEN Compact, Oxoid, Thermo Scientific, Dardilly, France). MALDI-TOF mass spectrometry (MS) protein analysis was carried out using a Microflex spectrometer^18^ (Bruker Daltonics, Bremen, Germany). Strain Marseille-P2911 spectra were imported into the MALDI BioTyper software (version 2.0, Bruker) and analysed by standard pattern matching (with default parameter settings). Interpretation of the scores was performed as previously described^19^.

Moreover, the 16S rRNA gene was sequenced using the fD1-rP2 primer pairs as previously described^20^, using a 3130-XL sequencer (Applied Biosciences, Saint Aubin, France). A phylogenetic tree was obtained using the Maximum Likelihood method and Kimura 2-parameter within the MEGA 7 software^21^. Several growth temperatures (20, 28, 37, 45 and 55 °C) on 5% sheep blood-enriched Columbia agar medium (BioMérieux, Marcy l’Etoile, France) were tested. Growth of strain Marseille-P2911 was tested under different atmospheres (anaerobic, aerobic and microaerophilic (CampyGEN, Oxoid). API ZYM and API 50CH strips (BioMérieux) were used to evaluate the biochemical properties of the strain test according to the manufacturer’s instructions. All API experiments were performed under anaerobic conditions. Using API 50CH, API 20E and API ZYM strips, strain Marseille-P2911 was incubated for 48, 24 and 4 hours, respectively. The standard disc method was applied for antimicrobial susceptibility testing according to the French Microbiology Society^13^. Finally, cellular fatty acid methyl ester (FAME) analysis was performed by GC/MS. Two samples were prepared with approximately 18 mg of bacterial biomass per tube harvested from several culture plates. Briefly, fatty acid methyl esters were separated using an Elite 5-MS column and monitored by mass spectrometry (Clarus 500 - SQ 8S, Perkin Elmer, Courtaboeuf, France)^22^. GC/MS analyses were carried out as described before^23^. Spectral database search was performed using MS Search 2.0 operated with the Standard Reference Database 1 A (NIST, Gaithersburg, USA) and the FAMEs mass spectral database (Wiley, Chichester, UK). For transmission electronic microscopy, detection formvar-coated grids were dropped onto a 40 μL bacterial suspension before incubation at 37 °C for 30 minutes. Then, the grids were incubated on 1% ammonium molybdate for 10 seconds, dried on blotting paper and finally observed using a Tecnai G20 transmission electron microscope (FEI, Limeil-Brevannes, France) at an operating voltage of 60 Kv. All methods were performed in accordance with the relevant guidelines and regulations.

### Extraction and genome sequencing

After a pretreatement by lysozyme incubation at 37 °C for 2 hours, DNA of strain Marseille-P2911 was extracted using an EZ1 biorobot (Qiagen) with the EZ1 DNA Tissue kit. The elution volume was 50 µL. Genomic DNA (gDNA) was quantified by a Qubit assay with the high sensitivity kit (Life technologies, Carlsbad, CA, USA) at 92.6 ng/µl. Genomic DNA was sequenced on a MiSeq sequencer (Illumina Inc, San Diego, CA, USA) with the Mate Pair strategy. The gDNA was barcoded in order to be mixed with 11 others projects with the Nextera Mate Pair sample prep kit (Illumina). The Mate Pair library was prepared with 1.5 µg of genomic DNA using the Nextera Mate Pair Illumina guide. The gDNA sample was simultaneously fragmented and tagged with a Mate Pair junction adapter. The pattern of the fragmentation was validated on an Agilent 2100 BioAnalyzer (Agilent Technologies Inc, Santa Clara, CA, USA) with a DNA 7500 labchip. DNA fragments ranged in size from 1.5 kb up to 11 kb with an optimal size at 8.4 kb. No size selection was performed and 600 ng of tagmented fragments were circularized. The circularized DNA was mechanically sheared to small fragments with an optimal at 706 bp on the Covaris device S2 in microtubes (Covaris, Woburn, MA, USA). The library profile was visualized on a High Sensitivity Bioanalyzer LabChip (Agilent Technologies Inc, Santa Clara, CA, USA) and the final concentration library was measured at 13.256 nmol/l. The libraries were normalized at 2 nM after a denaturation step and dilution at 15 pM, loaded onto the reagent cartridge and then onto the instrument along with the flow cell. Automated cluster generation and sequencing run were performed in a single 39-hours run in a 2 × 251-bp format. Total information of 8.3 gb was obtained from a 910 K/mm^2^ cluster density with a cluster passing quality control filters of 92.8% (16,316,000 passing filter paired reads). Within this run, the index representation for strain Marseille-P2911 was determined to 8.4%. The 688,244 paired reads were quality-checked using FastQC, trimmed using Trimmomatic version 0.36.6^24^ and assembled in two scaffolds using the SPAdes version 3.5.0 software^25^. The option “careful” was used in order to reduce the number of mismatches and short indels. Default parameters were applied for k values, i.e., k-mer values of 127, 99, 77, 55, 33, and 21. SSPACE^26^ and GapFiller^27^ were used to combine contigs, using default parameters^28,29^.

### Genome annotation and genome comparison

The genome was annotated as previously described^19^. In addition, we used the Genome-to Genome Distance Calculator (GGDC) web server available at (http://ggdc.dsmz.de) to estimate the overall similarity among the compared genomes and to replace the wet-lab DNA–DNA hybridization (DDH) by a digital DDH (dDDH)^30,31^. Average nucleotide identity analysis was also estimated using the orthoANI^32^ and MAGI^33^ softwares. Antibiotic resistance genes (ARG) were searched using the ARG-ANNOT database and Bio-Edit interface^34^. Assembled sequences were searched against the ARG-ANNOT database under moderately stringent conditions (e-value of 10^−5^) for the in silico ARG prediction. These putative ARGs were further confirmed through a BLAST search against non-redundant (nr) database in GenBank.

The presence of pathogenesis-related proteins was investigated using PathogeneFinder 1.1^35^. Finally, predicted protein sequences of strain Marseille-P2911 were used as queries to search the NCBI GenBank non-redundant protein sequence database. These results were formatted to generate network of protein sequences using the Cytoscape tool^36^.

## Supplementary information

Supplementary data
