# Supplementary material for: Colibacter massiliensis gen. nov. sp. nov., a novel Gram-stain-positive anaerobic diplococcal bacterium, isolated from the human left colon
Source: Sci Rep. 2019 Nov 20;9:17199. doi: 10.1038/s41598-019-53791-1 (PMC6868274; doi:10.1038/s41598-019-53791-1)
Supplement: Supplementary file 1 — Supplementary data [file 41598_2019_53791_MOESM1_ESM.docx]

**Title: *Colibacter massiliensis* gen. nov. sp. nov., a novel Gram-stain-positive anaerobic diplococcal bacterium, isolated from the human left colon**

**Authors:** Hussein ANANI^1,2^, Rita ABOU ABDALLAH^1,2^, May KHODER^2,3^, Anthony FONTANINI^2,3^, Morgane MAILHE^2,3^, Davide RICABONI^2,3^, Didier RAOULT^2, 3,4^, Pierre-Edouard FOURNIER^1,2^*

**Supplementary data**

**Figure:**

**
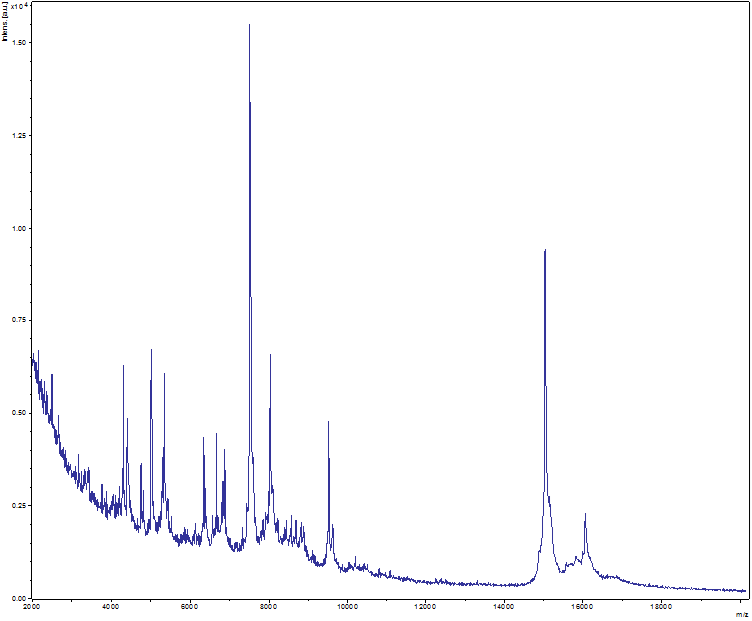
**

**Fig. S1** Reference mass spectrum from strain Marseille-P2911^T^.

**Tables:**

| **Fatty acids** | **Name** | **Mean relative % (a)** |
| --- | --- | --- |
| 12:0 | Dodecanoic acid | 25.3±1.7 |
| 16:0 | Hexadecanoic acid | 19.5±1.0 |
| 14:0 | Tetradecanoic acid | 13.7±0.6 |
| 14:0 3-OH | 3-hydroxy-Tetradecanoic acid | 9.4±1.4 |
| 18:1n9 | 9-Octadecenoic acid | 8.1±0.4 |
| 16:1n7 | 9-Hexadecenoic acid | 7.2±0.7 |
| 18:0 | Octadecanoic acid | 4.2±0.7 |
| 5:0 iso | 3-methyl-butanoic acid | 3.7±0.4 |
| 13:0 iso | 11-methyl-Dodecanoic acid | 2.7±0.4 |
| 15:0 iso | 13-methyl-Tetradecanoic acid | 2.3±0.7 |
| 17:0 iso | 15-methyl-Hexadecanoic acid | 1.7±0.3 |
| 16:1n5 | 11-Hexadecenoic acid | 1.3±0.6 |
| 18:2n6 | 9,12-Octadecadienoic acid | TR |
| 15:0 | Pentadecanoic acid | TR |

**Table S1** Cellular fatty acids composition (%)
^a^Mean peak area percentage ; TR = trace amounts < 1%

| Code | Value | % value | Description |
| --- | --- | --- | --- |
| J | 136 | 10.1 | Translation |
| A | 0 | 0 | RNA processing and modification |
| K | 63 | 4.67 | Transcription |
| L | 87 | 6.44 | Replication, recombination and repair |
| B | 0 | 0 | Chromatin structure and dynamics |
| D | 19 | 1.4 | Cell cycle control, mitosis and meiosis |
| Y | 0 | 0 | Nuclear structure |
| V | 22 | 1.63 | Defense mechanisms |
| T | 19 | 1.4 | Signal transduction mechanisms |
| M | 85 | 6.29 | Cell wall/membrane biogenesis |
| N | 0 | 0 | Cell motility |
| Z | 0 | 0 | Cytoskeleton |
| W | 0 | 0 | Extracellular structures |
| U | 23 | 1.7 | Intracellular trafficking and secretion |
| O | 44 | 3.26 | Posttranslational modification, protein turnover, chaperones |
| X | 0 | 0 | Mobilome: prophages, transposons |
| C | 106 | 7.85 | Energy production and conversion |
| G | 47 | 3.5 | Carbohydrate transport and metabolism |
| E | 111 | 8.22 | Amino acid transport and metabolism |
| F | 44 | 3.26 | Nucleotide transport and metabolism |
| H | 70 | 5.18 | Coenzyme transport and metabolism |
| I | 33 | 2.44 | Lipid transport and metabolism |
| P | 74 | 5.48 | Inorganic ion transport and metabolism |
| Q | 10 | 0.74 | Secondary metabolites biosynthesis, transport and catabolism |
| R | 125 | 9.26 | General function prediction only |
| S | 112 | 8.29 | Function unknown |
| - | 120 | 8.89 | Not in COGs |

**Table S2** Number of genes associated with the 25 general COG functional categories.

| Type strains | Size (Mb) | GC % | Gene content |
| --- | --- | --- | --- |
| *Colibacter massiliensis* | 1.72 | 50.2 | 1,655 |
| *Anaeroglobus geminatus* | 1.8 | 49 | 1,821 |
| *Dialister microaerophilus* | 1.28 | 35.4 | 1,237 |
| *Megasphaera paucivorans* | 2.91 | 40.2 | 2,810 |
| *Megasphaera micronuciformis* | 1.77 | 45.4 | 1,769 |
| *Megasphaera cerevisiae* | 3.24 | 44.8 | 3,152 |
| *Megasphaera elsdenii* | 2.5 | 52.8 | 2,366 |
| *Megasphaera massiliensis* | 2.74 | 50.2 | 2,576 |
| *Megasphaera hexanoica* | 2.88 | 49 | 2,787 |

**Table S3** Genomic comparison (sequence size, G+C contents and gene content) of *C. massiliensis* gen. nov. sp. nov. with the 8 most closely related bacterial taxa type strain for which genomes sequences were available.

|  | **CM** | **MP** | **ME** | **MMa** | **MMi** | **MC** | **DM** | **MH** | **AG** |
| --- | --- | --- | --- | --- | --- | --- | --- | --- | --- |
|  |  |  |  |  |  |  |  |  |  |
| **CM** | **100** | 21.50±4.7 | 20.30±4.6 | 18.10±4.5 | 18.20±4.5 | 19.90±4.6 | 27.10±4.8 | 22.00±4.7 | 19.10±4.5 |
| **MP** |  | **100** | 19.70±4.6 | 17.70±4.5 | 21.30±4.7 | 23.10±4.7 | 30.50±4.9 | 20.10±4.7 | 21.40±4.7 |
| **ME** |  |  | **100** | 24.30±4.8 | 26.00±4.8 | 19.20±4.6 | 28.00±4.9 | 23.70±4.8 | 21.10±4.7 |
| **MMa** |  |  |  | **100** | 20.00±4.6 | 18.30±4.5 | 24.90±4.8 | 20.20±4.6 | 18.50±4.5 |
| **MMi** |  |  |  |  | **100** | 21.70±4.7 | 28.60±4.8 | 24.80±4.8 | 18.20±4.5 |
| **MC** |  |  |  |  |  | **100** | 29.00±4.9 | 19.50±4.6 | 19.20±4.6 |
| **DM** |  |  |  |  |  |  | **100** | 27.80±4.8 | 25.30±4.8 |
| **MH** |  |  |  |  |  |  |  | **100** | 19.10±4.5 |
| **AG** |  |  |  |  |  |  |  |  | **100** |

**Table S4** dDDH values obtained by comparison of all studied genomes. *Colibacter massiliensis (CM)*, *Anaeroglobus geminatus (AG), Dialister micraerophilus (DM), Megasphaera paucivorans (MP), Megasphaera micronuciformis (MMi), Megasphaera cerevisiae (MC), Megasphaera elsdenii (ME), Megasphaera massiliensis (MMa)* and *Megasphaera hexanoica (MH)*.

dDDH digital DNA-DNA hybridization
